# Dynamics of d-serine reflected the recovery course of a patient with rapidly progressive glomerulonephritis

**DOI:** 10.1007/s13730-019-00411-6

**Published:** 2019-07-29

**Authors:** Atsushi Hesaka, Keiko Yasuda, Shinsuke Sakai, Hiroaki Yonishi, Tomoko Namba-Hamano, Atsushi Takahashi, Masayuki Mizui, Kenji Hamase, Rakan Matsui, Masashi Mita, Masaru Horio, Yoshitaka Isaka, Tomonori Kimura

**Affiliations:** 1 KAGAMI Project, National Institute of Biomedical Innovation, Health and Nutrition (NIBIOHN), Ibaraki, Osaka, Japan; 2 Reverse Translational Research Project, Center for Rare Disease Research, National Institute of Biomedical Innovation, Health and Nutrition (NIBIOHN), Ibaraki, Osaka, Japan; 3 Department of Nephrology, Osaka University Graduate School of Medicine, Suita, Osaka, Japan; 4 Graduate School of Pharmaceutical Sciences, Kyushu University, Fukuoka, Japan; 5 Shiseido Co., Ltd., Tokyo, Japan; 6 Department of Functional Diagnostic Science, Osaka University Graduate School of Medicine, Suita, Osaka, Japan

**Keywords:** d-serine, Rapidly progressive glomerulonephritis (RPGN), Systemic lupus erythematosus (SLE), Acute kidney injury (AKI), Fractional excretion (FE)

## Abstract

We experienced a case of a 36-year-old female with rapidly progressive glomerulonephritis (RPGN) due to anti-neutrophil cytoplasmic antibody (ANCA)-associated nephritis and systemic lupus erythematosus (SLE) nephritis. Chiral amino acid metabolomics revealed a prominent profile of d-serine in this patient. At the fulminant period of RPGN, the level of plasma d-serine, a potential biomarker in CKD that reflects actual glomerular filtration ratio (GFR), was extremely high. On the other hand, urinary fractional excretion (FE) of d-serine, which was usually much higher than that of l-isoform, was 0% in this patient. These abnormal d-serine profiles normalized in response to the intensive treatment. Normalizations of blood d-serine levels were in parallel with those of blood creatinine levels and potentially reflect the recovery of GFR. FE of d-serine increased transiently before the normalization of d-serine profile, suggesting that kidney promotes urinary excretion of d-serine for the normalization of plasma d-serine level. These unexplored clinical features of d-serine well reflected the clinical course of this patient. Blood d-serine level can also serve as a biomarker in acute kidney injury (AKI) or RPGN, and, in combination with FE of d-serine, may render the clinical practitioners to judge the efficacy of intensive treatments.

**Electronic supplementary material:**

The online version of this article (10.1007/s13730-019-00411-6) contains supplementary material, which is available to authorized users.

## Introduction

d-Amino acids, long-term undetected enantiomers of l-amino acids [1–3], are now emerging as potential biomarkers for several diseases including kidney diseases [4, 5]. In spite of their trace amount, d-serine does exist in human body [4], and plasma d-serine is now standing out with its usefulness in the estimation of kidney function, glomerular filtration ratio (GFR) [5], and in the prediction of the prognoses of the patients with chronic kidney disease (CKD) [4]. Additionally, urinary fractional excretion (FE) of d-serine turned out to have an association with the presence of kidney diseases [5]. These features of d-serine would potentiate the comprehensive management of CKD.

In light of clinical application, the question if the intra-body dynamics of d-serine reflect the disease course of kidney diseases arises. For example, do the profiles of d-serine reflect worsening or recovery phase of kidney injury? Based on the fact that the profiles of d-serine well correlate with GFR and are associated with the presence of kidney diseases, the profiles of d-serine may sensitively respond to the treatment of kidney diseases. If this is the case, d-serine can be utilized as biomarkers that can also reflect the effects of therapy.

We experienced a case of systemic lupus erythematosus (SLE), who had undergone a severe course of rapid progressive glomerular nephritis that responded well to the intensive care. We report the informative profile of d-serine of this case.

### Concise description of this case

Full clinical course of this case is described in the supplemental file. In brief, this case is a 36-year-old woman presented with RPGN. Laboratory test at kidney biopsy showed acute worsening of serum creatinine to 1032 μmol/L, high level of urinary protein (4 g/gCre), strong anemia (blood hemoglobin, 4.6 g/dL), normal levels of complements (C3, 88 mg/dL; C4, 21 mg/dL), positive anti-DNA antibody (13.0 IU/mL), and positive P-ANCA (182.0 U/mL). Kidney biopsy identified crescentic glomerulonephritis, potentially associated with ANCA, and SLE nephritis class V.

A series of plasma exchange was initiated followed by prednisolone pulse, oral prednisolone, intravenous cyclophosphamide, and mycophenolate mofetil. In response to these therapies, serum creatinine level improved to 63.65 μmol/L, while urinary protein level persisted. Followed-up kidney biopsy showed regression of cellular crescents in glomeruli, while 30% of glomeruli were globally sclerosed and capillary thickenings persisted.

### Dynamics of d-amino acids in this patient during the recovery phase

**Fig. 1:**
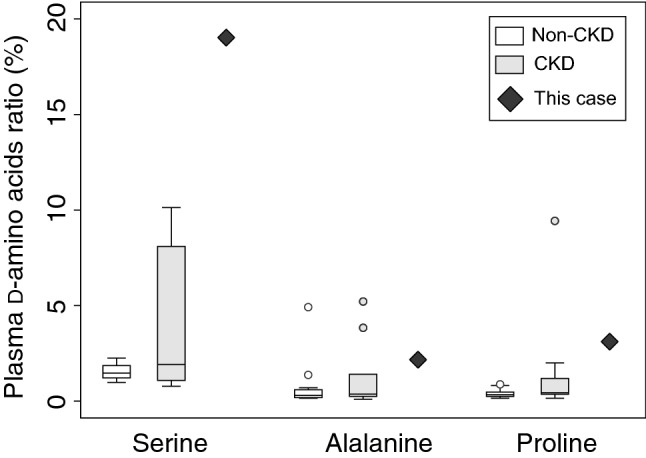
Plasma d-amino acids per total amino acids (*D* ratio [%]) of this patient on admission. Reference data (non-CKD and CKD) are from [5]. *CKD* chronic kidney disease

We examined the levels of d-amino acids throughout the recovery phase of RPGN due to SLE nephritis in this patient. Blood levels of d-amino acids at acute phase (just after admission to the hospital) were extraordinary; blood level of d-serine was extremely high and comprised 19% of whole blood serine (ranges of non-CKD were 1.22–1.85%, Fig. 1 and Supplementary Tables S1 and S2). d-Alanine and d-proline, which are often detected in normal population, were also high in this patient; these d-amino acids comprised 2.2% and 3.1% of each amino acid, respectively.

Among d-amino acids, d-serine was demonstrated to reflect GFR [5]. In this patient, blood levels of d-serine decreased in response to the treatment and these decreases were in parallel with those of blood creatinine levels. Finally, blood levels of d-serine further decreased to the normal ranges.

**Fig. 2:**
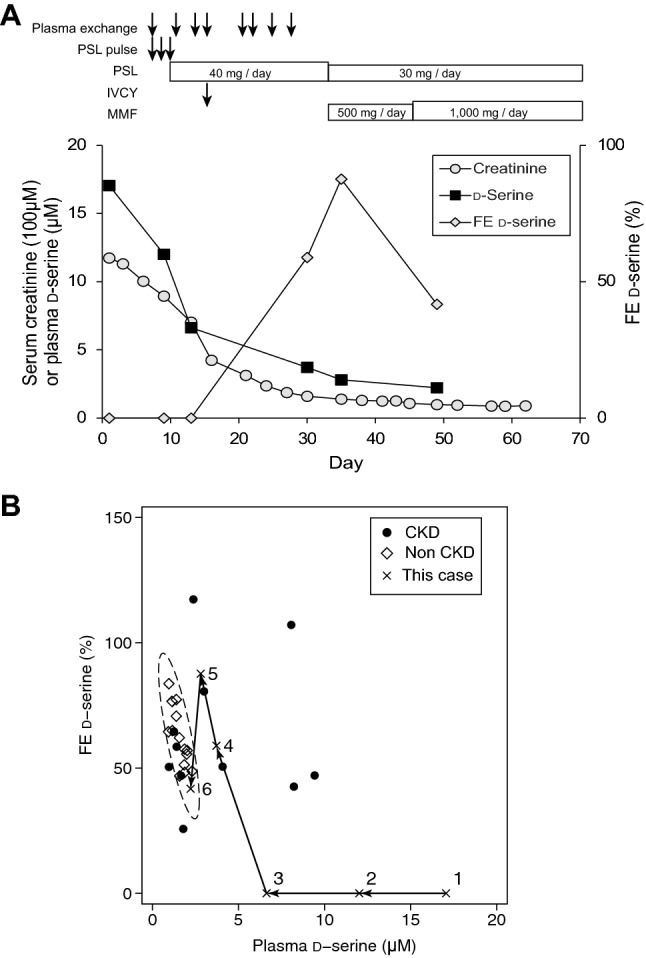
Dynamics of d-serine during the recovery phase of this patient. **a** Clinical course of this patient. *PSL* prednisolone, *IVCY* intravenous cyclophosphamide, *MMF* mycophenolate mofetil. **b** Dynamics of d-serine were plotted on a scatter plot with reference data [5]. The eclipse represents 95% confidence interval of non-CKD population. Each numbered dot of this patient reflected the following clinical course: 1, day 1 (on admission); 2, day 9 (before the first plasma exchange); 3, day 13 (before the second plasma exchange); 4, day 30 (after 8 sessions of plasma exchange); 5, day 35 (after IVCY); 6, day 49 (after the initial treatment just before the discharge)

Since FE of d-amino acids was reported to reflect disease profile in CKD [5], we also examined FE of d-amino acids during the clinical course of this patient (Fig. 2a, b). FE of d-serine was calculated as follows: urinary d-serine times blood creatinine divided by urinary creatinine and blood d-serine. FE is the ratio of a substrate filtered by the kidney glomerular that is excreted in the urine. At the initial course of this case, FE of d-serine was 0% due to no excretion of d-serine into the urine. FE of d-serine usually is reported to take wide ranges similarly in both normal and CKD population, whereas 0% of FE of d-serine was completely out of those ranges. FE of d-serine remained 0% until the end of plasma exchange sessions. During the recovery phase, FE of d-serine increased transiently even though the blood levels of both creatinine and d-serine were still high, reflecting the increased excretion of d-serine at this stage. After the series of treatment described here, blood level and FE of d-serine finally normalized to the profile compatible with normal population.

## Discussion

We experienced a case of RPGN with dynamic changes in both blood level and FE of d-serine during the recovery phase. At the fulminant phase of RPGN, the blood levels of d-serine were extremely high. On the other hand, FE of d-serine, which was usually much higher than that of l-isoform, was 0% in this patient. These abnormal d-serine profiles normalized in response to the intensive treatment. Normalizations of the blood levels of d-serine were in parallel with those of creatinine. During the recovery phase, FE of d-serine increased transiently and exceeded the normal ranges, followed by a drop in the normal ranges. This dynamic d-serine profile well reflected the clinical course of this patient.

Plasma d-serine may serve as a sensitive marker for AKI and RPGN. Our previous study revealed that d-serine reflects kidney function, GFR, and the presence of CKD [5]. d-Amino acids were known to be handled by kidney; after glomerular filtration, kidney reabsorbs amino acids at proximal tubules with chiral selectivity. In CKD patients with decreased GFR, blood levels of d-serine increased due to less glomerular filtration of d-serine. In line with this study, plasma level of d-serine also reflected recovery phase of RPGN. The abnormally high blood level of d-serine resolved with the treatment of SLE, and the longitudinal course of plasma level of d-serine was in parallel with serum level of creatinine, reflecting the recovery of GFR. Plasma d-serine may be applicable in examining treatment effects in AKI. Additionally, plasma d-serine may also be useful in detecting AKI and RPGN. The presence of a time lag between GFR decrease and blood creatinine increase in AKI is widely known, and whether blood d-serine responds to AKI promptly or not needs to be examined. Once nephrologists all over the world noticed the value of d-serine and started using it, we believe the cost will be reduced very rapidly, and d-serine will be available for daily clinics soon.

On the other hand, the reabsorption of d-serine is sensitive to the presence of CKD [5]. FE of d-serine increases in some patients with normal ranges of blood d-serine level, suggesting that the increment of FE of d-serine is a compensatory mechanism to keep blood d-serine levels low. Another patient showed decreased FE of d-serine, possibly due to decreased GFR, which in turn increased the blood d-serine levels. Therefore, FE of d-serine proceeded the increase of plasma d-serine and turned out as a useful marker for detecting kidney diseases before the worsening of GFR.

This concept was exemplified in this patient; the kidney promotes urinary excretion of d-serine for the normalization of plasma d-serine level. Increase in FE of d-serine during the recovery phase may represent the efficacy of the treatment, a feature essential for the drug discovery in kidney diseases.

In summary, we experienced a case of RPGN due to SLE nephritis accompanied with dynamic profiles of d-serine during its recovery phase. Blood levels of both creatinine and d-serine normalized in response to the treatment, and transient increase in urinary FE of d-serine played a role in normalization. Blood d-serine can serve as a biomarker for AKI and RPGN by reflecting GFR. Those lines of thus-far unexplored course of d-serine suggest the robust utility as a biomarker to monitor disease activity of kidney diseases and the treatment effects.

## Electronic supplementary material

Below is the link to the electronic supplementary material.

Supplementary material 1 (PDF 1431 kb)

## References

[1] Hashimoto A, Nishikawa T, Hayashi T, Fujii N, Harada K, Oka T (1992). The presence of free d-serine in rat brain. FEBS Lett.

[2] Krebs HA (1935). Metabolism of amino-acids: deamination of amino-acids. Biochem J.

[3] Mothet JP, Parent AT, Wolosker H, Brady RO, Linden DJ, Ferris CD (2000). d-serine is an endogenous ligand for the glycine site of the *N*-methyl-d-aspartate receptor. Proc Natl Acad Sci USA.

[4] Kimura T, Hamase K, Miyoshi Y, Yamamoto R, Yasuda K, Mita M (2016). Chiral amino acid metabolomics for novel biomarker screening in the prognosis of chronic kidney disease. Sci Rep.

[5] Hesaka A, Sakai S, Hamase K, Ikeda T, Matsui R, Mita M (2019). d-Serine reflects kidney function and diseases. Sci Rep.

