# Supplementary material for: Dynamics of d-serine reflected the recovery course of a patient with rapidly progressive glomerulonephritis
Source: CEN Case Rep. 2019 Jul 29;8(4):297–300. doi: 10.1007/s13730-019-00411-6 (PMC6820815; doi:10.1007/s13730-019-00411-6)
Supplement: Supplementary file 1 — Supplementary material 1 (PDF 1431 kb) [file 13730_2019_411_MOESM1_ESM.pdf]

Supplementary Information

D-Serine reflects kidney function and diseases

Atsushi Hesaka, Keiko Yasuda, Shinsuke Sakai, Hiroaki Yonishi, Tomoko Namba, Atsushi Takahashi, Masayuki Mizui, Kenji Hamase, Rakan Matsui, Masashi Mita, Masaru Horio, Yoshitaka Isaka, and Tomonori Kimura

2 Supplementary Tables
1 Supplementary Figure
Full description of this case
Supplementary Methods

Supplementary Table 1. Chiral amino acid profile of this patient on admission.

Amino acid	Concentration (uM)	Reference	
		non-CKD	CKD
D-Ser	17.06	1.56 (1.15 - 1.87)	2.39 (1.55 - 6.08)
L-Ser	72.6	93.7 (89.6 - 112.6)	114.7 (106.0 - 123.6)
D-Ala	10.07	0.99 (0.71 - 1.82)	1.28 (0.74 - 2.86)
L-Ala	452.9	286.7 (255.6 - 368.0)	328.5 (309.3 - 457.2)
D-Pro	7.59	0.48 (0.33 - 0.62)	0.78 (0.50 - 1.57)
L-Pro	236.5	123.2 (105.1 - 159.1)	143.1 (140.0 - 201.0)

Values, μM. Reference data are from Hesaka A. et al [5].

Supplementary Table 2. Fractional excretion of chiral amino acid of this patient on admission.

Amino acid	FE (%)	Reference	
		non-CKD	CKD
D-Ser	0	62.1 (53.4 - 73.6)	50.5 (47.0 - 72.5)
L-Ser	0.21	1.30 (0.75 - 1.96)	1.21 (0.52 - 9.70)
D-Ala	39.9	20.7 (17.7 - 22.2)	22.8 (18.3 - 39.4)
L-Ala	9.6	0.28 (0.21 - 0.44)	0.40 (0.26 - 2.71)
D-Pro	7.1	ND	ND
L-Pro	0.53	0.026 (0.022 - 0.031)	0.056 (0.041 - 0.25)

Values, μM; ND, not determined. Reference data are from Hesaka A. et al [5].

Supplementary Figure 1

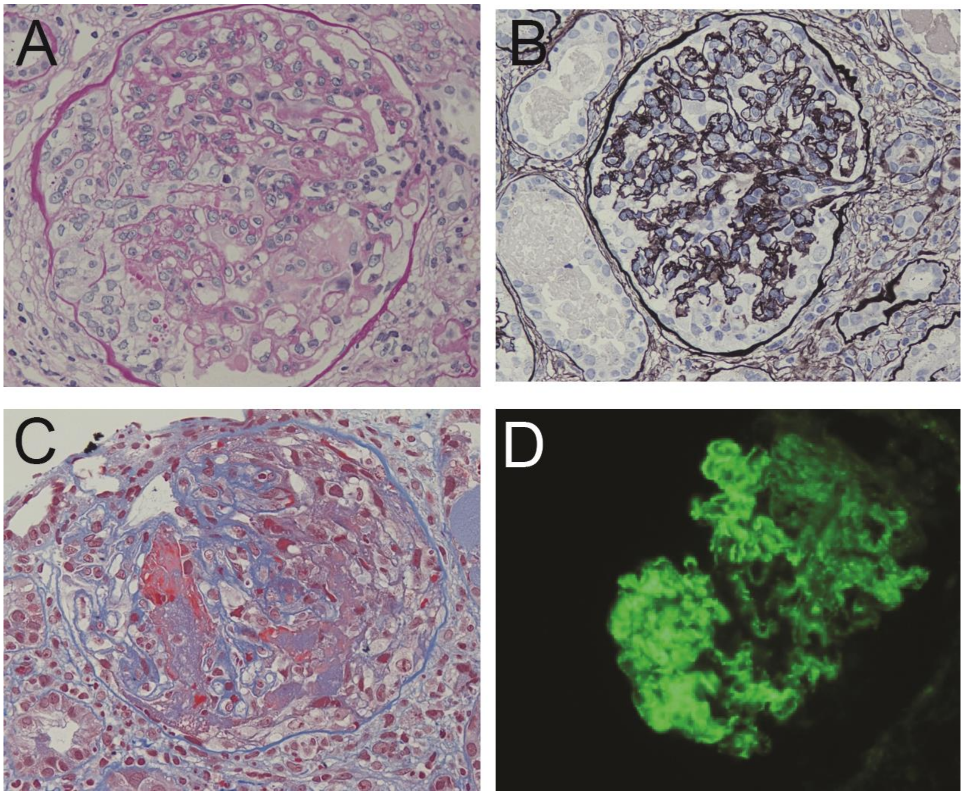

Supplementary Figure 1. Renal biopsy findings of this patient. (A) A glomerulus exhibits a cellular crescent. Periodic Acid Schiff (PAS) staining. (B) Periodic acid methenamine silver (PAM) staining shows thickening of basal membrane with spikes and double-contours. (C) Elastica-Masson staining shows the presence of a segmental fibrinoid necrosis. (D) Immunofluorescence staining for C3 shows fulminant deposits in the basal membrane. (Magnification, ×400).

Full description of this case

A 36-year old woman presented with rapid worsening of kidney function. 90 days prior to introduction to our hospital, her serum creatinine level was 50.39 μmol / L, but it worsened acutely to 1032 μmol / L. Urinary protein level increased from 0.5 g/gCre to 4 g/gCre. Melena and skin rash on the back of her hands emerged for a month. Having been pointed out for proteinuria with hematuria for 11 years, she underwent kidney biopsy 9 years ago. Laboratory test at that time showed decreased blood lymphocytes, positive urinary protein (1.5 g/gCre), decreased levels of blood complements, and positive for autoantibodies (anti-nuclear antibody and and-ds DNA antibody). She was diagnosed as having SLE nephritis class V, but she was untreated thereafter and underwent natural childbirth twice.

Her consciousness was clear, but she felt malaise with her face strikingly pale. Blood pressure, 122/65 mmHg; heart ratio, 64 bpm; percutaneous arterial oxygen saturation, 100% (room air); body temperature, 36.5C. Mouse ulcers, depilation, and retinal hemorrhage were pointed out. Neither abnormal lung sounds, heart sounds, nor leg edema were observed. Laboratory tests showed strong anemia (blood hemoglobin, 4.6 g/dL), normal levels of complements (C3, 88 mg/dL; C4, 21 mg/dL), positive anti-DNA antibody (13.0 IU/mL), and positive P-ANCA (182.0 U/mL). Rapid progressive glomerular nephritis was suspected.

Followed by the first session of plasma exchange, fine-needle kidney biopsy was performed. Most glomeruli (79 %) showed cellular crescents, and 13 % showed cellular-fibrillary crescents (Supplementary Figure 1). Glomerular capillaries were thickened with bubbling and spikes. No glomerular-sclerosis was observed. Interstitial regions showed moderate and diffuse infiltration of inflammatory cells, but fibrillation was only slightly. Tubular atrophy was focal and slight. Immunofluorescence staining revealed granular global glomerular capillary wall positivity for IgG, IgA, IgM, C3, C4, and C1q. Crescentic glomerulonephritis, potentially associated with ANCA, and SLE nephritis class V were diagnosed.

She was treated with subsequent sessions of plasma exchange, prednisolone pulse therapy (1g for 3 days) followed by oral prednisolone (40 mg/day), intermittent pulse intravenous cyclophosphamide therapy (500 mg/m^2) and mycophenolate mofetil (MMF, 500 mg/day, Figure 2A). In response to these therapy, serum creatinine level improved to 63.65 μmol / L, while urinary protein level persisted. Followed-up kidney biopsy showed regression of cellular crescents in glomeluri, while 30% of glomeruli were globally sclerosed and capillary thickenings persisted.

Supplemental Methods

Sample preparation.

Sample preparation from human plasma and urine was performed as previously described with modification [6,7]. In brief, 20-fold volumes of methanol were added to the sample and an aliquot (10 μL of the supernatant obtained from the methanol homogenate) was placed in a brown tube and used for NBD derivatization (0.5 μL of the plasma was used for the reaction). After drying the solution under reduced pressure, 20 μL of 200 mM sodium borate buffer (pH 8.0) and 5 μL of fluorescence labeling reagent (40 mM 4-fluoro-7-nitro-2,1,3-benzoxadiazole (NBD-F) in anhydrous MeCN) were added, then heated at 60°C for 2 min. An aqueous 0.1 % (v/v) TFA solution (75 μL) was added, and 2μL of the reaction mixture was subjected to 2D-HPLC.

Determination of amino acid enantiomers by 2D-HPLC.

The enantiomers of amino acids were quantified using the micro 2D-HPLC platform, as previously described [6,7]. In brief, the NBD-derivatives of the amino acids were separated using a reversed-phase column (monolithic ODS column, 0.53 mm i.d. × 1000 mm; provided by Shiseido, Tokyo, Japan) with the gradient elution using aqueous mobile phases containing MeCN, THF, and TFA. In order to separately determine the D- and L-forms, the fractions of the target amino acids were automatically collected using a multi-loop valve, and transferred to the enantioselective column (KSAACSP-001S or Sumichiral OA-3200, 1.5 mm i.d. × 250 mm; self-packed. Materials were obtained from Shiseido and Sumika Chemical Analysis Service, Osaka, Japan, respectively). The mobile phases are the mixed solution of MeOH-MeCN containing citric acid or formic acid, and the fluorescence detection of the NBD-amino acids was carried out at 530 nm with excitation at 470 nm. All the quantitative data were obtained by the fluorescence detection.

<u>Fractional excretion calculation.</u>

Fractional excretion (Fe, %) were calculated from clearance of substrate divided by that of creatinine. The formula is as follows.

$$Fe\ Substrate = \frac{Substrate\ clearance}{\text{Creatinine clearance}} = \frac{\text{Us x V / Ps}}{\text{Ucre x V / Pcre}}$$
$$= \frac{\text{Us x Pcre}}{\text{Ucre x Ps}}$$

, where Us and Ps represent urinary and plasma levels of substrate, respectively. Fractional excretion is the ratio of a substrate filtered by the kidney glomerular that is excreted in the urine. Fractional excretion reflects the excretion of substrates into the urine, the resultant of glomerular filtration and tubular reabsorption and / or secretion.

References

6. Hamase K, Miyoshi Y, Ueno K, et al. Simultaneous determination of hydrophilic amino acid enantiomers in mammalian tissues and physiological fluids applying a fully automated micro-two-dimensional high-performance liquid chromatographic concept. *J Chromatogr A.* 2010;1217(7):1056-1062.

7. Miyoshi Y, Hamase K, Tojo Y, Mita M, Konno R, Zaitsu K. Determination of D-serine and D-alanine in the tissues and physiological fluids of mice with various D-amino-acid oxidase activities using two-dimensional high-performance liquid chromatography with fluorescence detection. *J Chromatogr B.* 2009;877(24):2506-2512.
